# Comparative genomics reveals the molecular mechanism of salt adaptation for zoysiagrasses

**DOI:** 10.1186/s12870-022-03752-0

**Published:** 2022-07-21

**Authors:** Wei Wang, An Shao, Xiao Xu, Shugao Fan, Jinmin Fu

**Affiliations:** Coastal Salinity Tolerant Grass Engineering and Technology Research Center, Ludong University, Yantai, Shandong China

**Keywords:** *Zoysia*, Comparative analysis, Whole-genome duplication, Salt adaptation

## Abstract

**Background:**

Zoysiagrass (*Zoysia* spp.) is a warm-season turfgrass. It is widely used as turfgrasses throughout the world, offers good turf qualities, including salt tolerance, resistance to drought and heat. However, the underlying genetic mechanism of zoysiagrass responsive to salt stress remains largely unexplored.

**Results:**

In present study, we performed a whole-genome comparative analysis for ten plant genomes. Evolutionary analysis revealed that Chloridoideae diverged from Panicoideae approximately 33.7 million years ago (Mya), and the phylogenetic relationship among three zoysiagrasses species suggested that *Zoysia matrella* may represent an interspecific hybrid between *Zoysia japonica* and *Zoysia pacifica*. Genomic synteny indicated that *Zoysia* underwent a genus-specific whole-genome duplication (WGD) event approximately 20.8 Mya. The expression bais of homologous genes between the two subgenomes suggested that the B subgenome of *Z. japonica* contributes to salt tolerance. In additon, comparative genomic analyses revealed that the salt adaptation of *Zoysia* is likely attributable to the expanded cytochrome P450 and ABA biosynthetic gene families. Furthermore, we further found that many duplicated genes from the extra WGD event exhibited distinct functional divergence in response to salt stress using transcriptomic analysis, suggesting that this WGD event contributed to strong resistance to salt stress.

**Conclusions:**

Here, our results revealed that expanded cytochrome P450 and ABA biosynthetic gene families, and many of those duplicated genes from recent *zoysia*-specific WGD event contributed to salt adaptation of zoysiagrass, which provided insight into the genetic underpinning of salt adaptation and valuable information for further studies on salt stress-related traits in *Zoysia*.

**Supplementary Information:**

The online version contains supplementary material available at 10.1186/s12870-022-03752-0.

## Background

Soil salinity is one of the major environmental factors that limits crop yield and plant distribution worldwide [1, 2]. It has been reported that more than 20% of global irrigated areas are seriously affected by salinity, and the situation is worsening [3, 4]. High concentrations of salt trigger osmotic stress and ion toxicity and possibly cause oxidative stress and a series of secondary stresses [2]. The detrimental effects of these stresses on plants can be observed as the death of plants and/or decreases in productivity [5]. Growing evidence indicates that some forage grass and turfgrass species are more tolerant to salt stress than many cultivated crop varieties, and some of them can be divided into halophytes [6]. Therefore, understanding the molecular mechanisms underlying salt stress in halophytes may provide valuable information for the management and usage of these special wild resources [7].

Zoysiagrass (*Zoysia* spp. Willd.) is a genus of perennial plants belonging to the family Poaceae, subfamily Chloridoideae, tribe Zoysieae and is recognized as an excellent warm-season turfgrass with salt and drought tolerance worldwide [8]. *Zoysia* comprises 11 species, all of which are allotetraploids (2*n* = 4*x* = 40), and *Z. japonica*, *Z. matrella* and *Z. pacifica* are considered to be the most economically important and have been widely used as turfgrasses throughout the world [9, 10]. Numerous studies have assessed the effects of salinity and revealed that zoysiagrass can resist injury under 1.0% salt solution and are considered to be the most salt tolerant of the C4 grass species in the family *Poaceae* [7, 11]. In particular, *Z. japonica* and *Z. matrella* are distinctly tolerant to salt stress and are defined as halophytes [11, 12].

Previous studies have mainly focused on the evaluation of salt tolerance and the physiological mechanisms governing salt tolerance [10]. Several studies on zoysiagrasses have attempted to trace their evolutionary history and explore the molecular mechanism of salt tolerance. Synteny comparisons between *Z. japonica* pseudomolecules and the *Sorghum bicolor* or *Oryza sativa* genome indicated that *Z. japonica* underwent chromosome rearrangement events [9], these rearrangements eventually led to the ancestor of zoysiagrass undergo two nested chromosomal fusion (NCF) events to reduce the base chromosome number from the n = 12 intermediate ancestral genome to 10, which consistent with the hypothesis proposed previously that nested chromosome fusion is the dominant mechanism of reduction of chromosome number in grasses [13, 14]. Subsequently, zoysiagrass inherited equivalent basic chromosome number from the common ancestor of Chloridoideae and evolved into different zoysiagrasses. Genetic variation and population structure analysis among 248 *Zoysia* accessions suggested that *Z. matrella* might represent an interspecific hybrid between *Z. japonica* and *Z. pacifica* [15]. Dissecting the genetics of salt tolerance founded that a significant quantitative trait locus (qSCW-1) for the reduced percentage of dry shoot clipping weight under salt stress was detected at 44.1 cM on LG5 and explained 65% of the total variation using the constructed genetic linkage map between salt-tolerant Z105 and salt-sensitive Z061 [16], and the differences in sequence polymorphisms of the *ANAC102* and *STO/BBX24* genes may contribute to the variation in salt tolerance levels among these three *Zoysia* species [9]. Plants living in harsh environments often exhibit specialized morphological and physiological adaptations to abiotic stresses [17], and through long-term adaptation to the extremely coastal salinity environment, zoysiagrass has developed a considerable level of resistance to salt and is expected to leave genetic and genomic signatures. However, due to a lack of in-depth studies on *Zoysia*, the evolutionary relationships among zoysiagrass species is still lack of persuasive evidence from the whole genome, and the genetic basis of adaptation to salt stress also still stayed at the signle candidated gene level, therefore, much more work is needed to clarify these topics to gain new insights into the evolution of salt stress.

In this study, to better understand the molecular evolutionary mechanisms underlying salt stress in *Zoysia*, we identified genomic signatures of adaptive evolution for three *Zoysia* species, including *Zoysia* members that underwent shared whole-genome duplication (WGD) events after divergence from the *Oropetium thomaeum* lineage, and the expanded cytochrome P450 and the abscisic acid (ABA) biosynthetic pathway contributed to the adaptation of *Zoysia* to saline environments. In addition, the results of homolog expression patterns and subgenome dominance suggested that the B subgenome may play a more important role in salt stress adaptation in *Z. japonica* than the A subgenome. Finally, transcriptomic analysis suggested that many duplicated genes from the extra WGD event exhibited distinct functional divergence and were probably responsible for salt adaptation. Our study will offer the thread of the adaptive genetic mechanisms in salt stress adaptation and provide valuable information for further studies on salt stress-related traits in *Zoysia*.

## Results

### Analysis of gene family dynamics in Zoysiagrass genomes

**Fig. 1:**
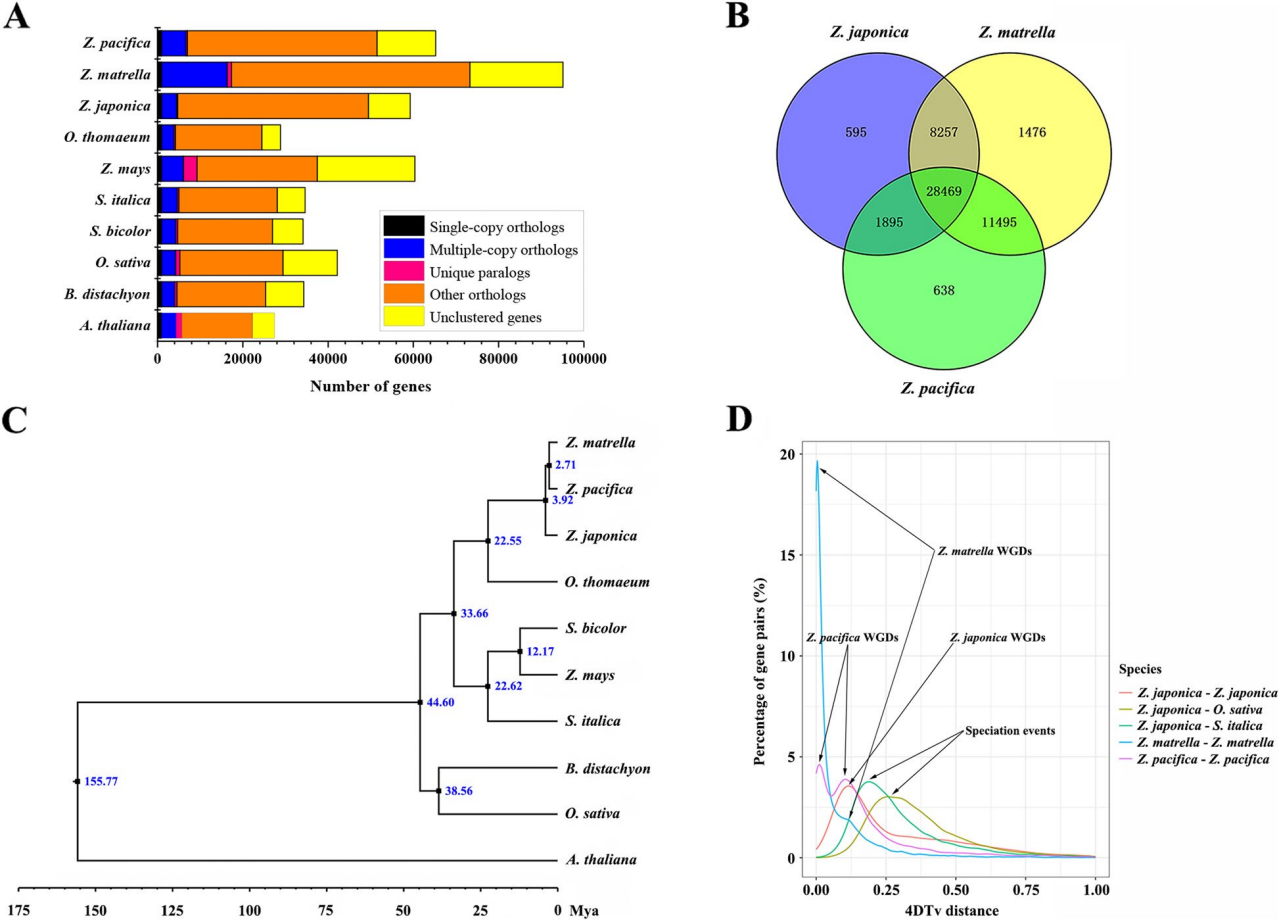
Evolutionary and comparative genomic analyses. (**A**) Gene categories used from all the species. (**B**) Venn diagram of shared orthologous gene families in three species. The number of gene families is listed for each component. (**C**) Phylogenetic relationships and times of divergence of three zoysiagrass with seven other plant species. Divergence times labelled in blue. (**D**) Distribution of 4DTv distance of syntenic blocks between and within Poaceae species

Ortholog clustering can be used to identify important patterns in gene conservation across diverse organisms and reveal species or lineage-specific sets of genes that are important for one species or group. To investigate the dynamic patterns of gene families among three Zoysiagrass species (*Z. japonica, Z. matrella* and *Z. pacifica*), genes in the genomes of nine Poaceae species and the model plant Arabidopsis were classified into groups of orthologous genes using OrthoMCL [18]. A total of 368,346 of the input protein sequences (76.5%, 481,420) were assigned to 64,947 orthogroups, and 7,495 and 10,404 orthogroup genes were identified in all ten species and nine grass genomes, respectively (Fig. 1A). Gene family clustering showed that 28,469 core gene families were shared by three zoysiagrass species, and 13,720 orthogroups were three zoysiagrass species-specific gene families containing 13,995, 18,583 and 14,628 genes for *Z. japonica, Z. matrella* and *Z. pacifica*, respectively (Fig. 1B). In addition, 228, 988 and 385 orthogroups containing 1,411, 1,994 and 793 genes were species-specific gene families for *Z. japonica*, *Z. matrella* and *Z. pacifica*, respectively (Additional file 1: Table S1).

### Phylogenetic tree construction and whole-genome duplications

To clarify the interrelated evolutionary histories among the three zoysiagrass species, a high-confidence phylogenetic tree of the ten species, including nine grass species and *A. thaliana* as an outgroup, was constructed using genes extracted from 963 single-copy gene families by a maximum likelihood method using RaxML software (Fig. 1C). The resulting phylogeny indicated that the *Oryza*- and *Sorghum*-*Zoysia* divergence times were estimated to be approximately 44.60 and 33.66 million years ago (Mya), respectively. Among the three *Zoysia* species, *Z. matrella* had a closer relationship with *Z. pacifica* than *Z. japonica*, and *Z. matrella* and *Z. pacifica* diverged from each other approximately 2.71 Mya, while *Z. japonica* had a divergence time of 3.92 Mya for *Z. matrella* or *Z. pacifica*, suggesting that climate change during the Pliocene might have driven the formation and differentiation of *Zoysia* species.

Synteny analysis using MCScanX revealed 347, 2,552 and 617 syntenic blocks within the genome of *Z. japonica*, *Z. matrella* and *Z. pacifica* and identified syntenic blocks between the genomes of *Z. japonica* and *O. sativa* and *Z. japonica* and *Setaria italica*. We calculated the transversion rate at the fourfold degenerate sites (4dTv) and synonymous substitutions per site (Ks) of paralogous and orthologous gene pairs in the *Zoysia* genome. The distribution of 4dTv indicated that whole-genome duplication (WGD) events occurred in *Zoysia* members after divergence between *Oryza*- and *Sorghum*-*Zoysia* (Fig. 1D). The distribution of synonymous substitutions per site (Ks) showed prominent orthologous peaks at Ks = 0.64 and 0.48 in *Z. japonica*-*O. sativa* and –*S. italica*, reflecting the divergence time between *O. sativa*- and *S. italica*-*Zoysia* dated to 49.2 and 36.9 Mya, respectively, which are consistent with the estimations based on the phylogenetic tree (Additional file 2: Fig. S1A and B). The paralogous gene pairs in the three *Zoysia* genomes showed one obvious peak with Ks = 0.27, revealing that a WGD event occurred approximately 20.8 Mya and was shared by three *Zoysia* members. The calculation of Ks for *Z. japonica* versus *O. thomaeum* further validated that this WGD event occurred after the split of *Z. japonica* and *O. thomaeum* (Additional file 2: Fig. S1C and D). In addition, the analysis also revealed a Ks peak specific to *Z. matrella* (Ks = 0.02) and *Z. pacifica* (Ks = 0.03), suggesting that species‐specific whole‐genome duplication events occurred after the split of *Z. matrella* and *Z. pacifica* (Fig. 1D).

### Genome-wide expression dominance

**Fig. 2:**
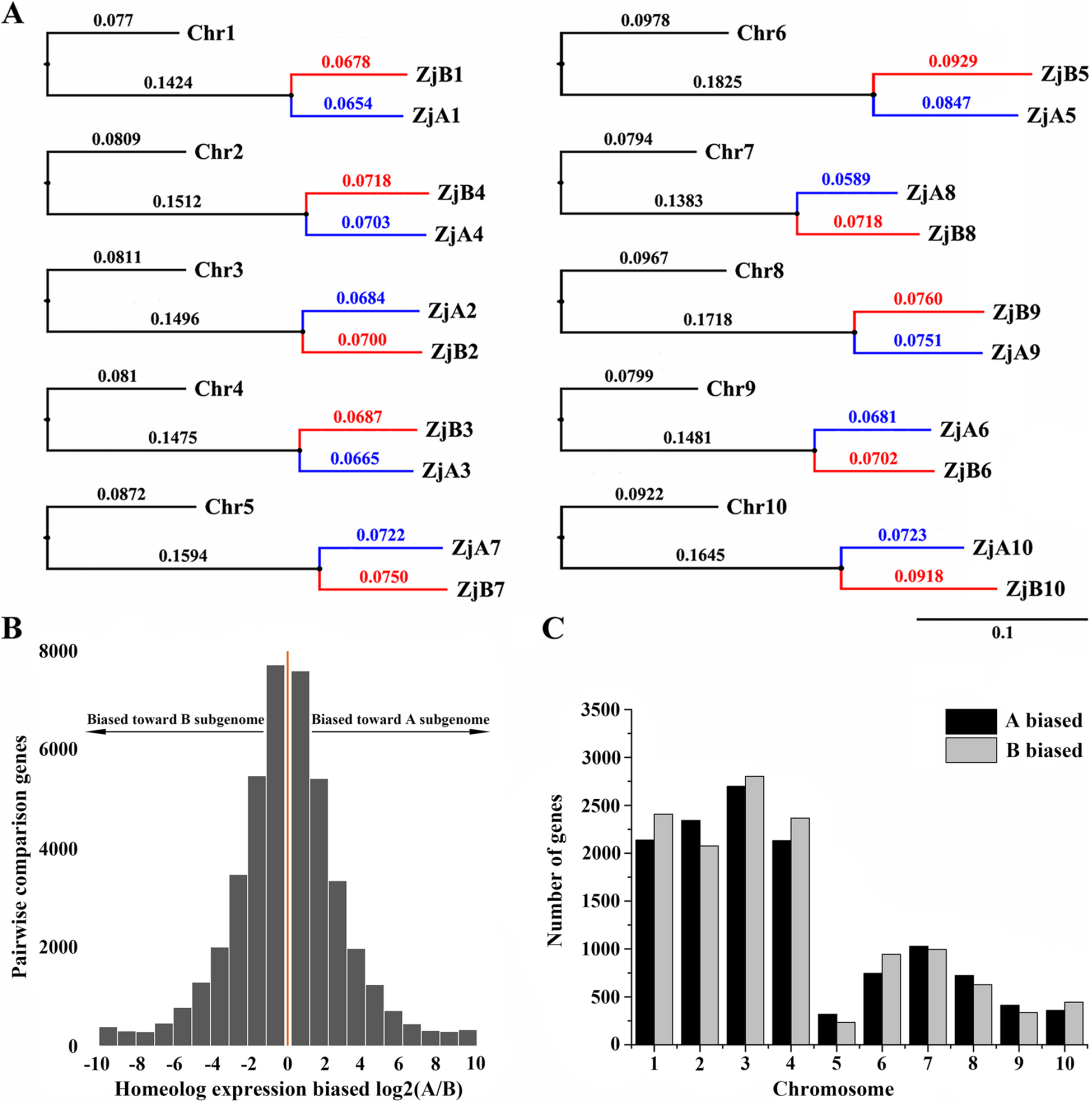
Homoeolog expression bias between the A and B subgenomes of *Z. japonica.* (**A**) Phylogenetic tree based on single copy orthologs shows chromosomal relationships between *Z. japonica* (Zj) and *O. thomaeum* (Ot). (**B**) The distribution of homoeolog expression bias (HEB) between all gene pairs in all tissues. An HEB > 0 indicates bias toward the A subgenome and a HEB < 0 indicates bias toward the B subgenome. (**C**) HEB in each of the ten pairs of chromosomes across all eight tissue types

High interchromosomal colinearity among the 20 pseudochromosomes strongly suggests the existence of subgenomes in *Z. japonica* (Additional file 3: Fig. S2A). To investigate the structure of the subgenomes, we conducted a phylogenomics study between *Z. japonica* pseudochromosomes and *O. thomaeum*, which has the closest phylogenetic relationship with *Z. japonica* (Fig. 1C). The obtained results showed that each *O. thomaeum* chromosome corresponded to a pair of *Z. japonica* pseudochromosomes, and ten pseudochromosomes 2, 3, 5, 8, 10, 12, 13, 15, 17 and 20 with a closer genetic relationship with the ten *O. thomaeum* were classified into the A subgenome, while the other ten pseudochromosomes were classified into the B subgenome (Fig. 2A and Additional file 3: Fig. S2B).

To investigate patterns of subgenome differentiation and dominance, we conducted transcriptional analyses of homologous genes in two distinct tissue types and three stages of progressive salt stress. In total, 11,315 syntenic gene pairs between the A and B subgenomes showed homologous expression bias (HEB) in at least one sample, with 50.6% showing biased expression toward homologs in the B subgenome (Fig. 2B). Although these results revealed the absence of significant global genome dominance between the A and B subgenomes, gene enrichment analysis suggested that more pathways were enriched in response to abiotic stimulus, hormones and other stress-response pathways for homologous expression bias from the B subgenome (Additional file 4: Table S2). In addition, the chromosomal distribution indicated that five pairs of chromosomes showed HEB toward the A subgenome, and the other five pairs of chromosomes contained more dominant homologs from the B subgenome. The difference in the numbers of homologs was not significant in homologous chromosomes (Fig. 2C).

### Gene family expansion and contraction

To reveal the genetic basis underlying the adaptive evolution of the three zoysiagrass phenotypes, we assessed the function of shared and species-specific gene families for the three zoysiagrass genomes. Gene Ontology (GO) enrichment analysis indicated that 28,469 core gene families shared by three zoysiagrass species were significantly enriched in the categories of biological regulation (GO:0,065,007), developmental process (GO:0,032,502) and response to light stimulus (GO:0,009,416) (Additional file 5: Table S3). A total of 13,720 orthogroups specific to the three zoysiagrass species were significantly overrepresented in signal transduction (GO:0,007,165), response to stress (GO:0,006,950), reproduction (GO:0,000,003), and response to hormones (GO:0,009,725) (Additional file 6: Table S4).

**Fig. 3:**
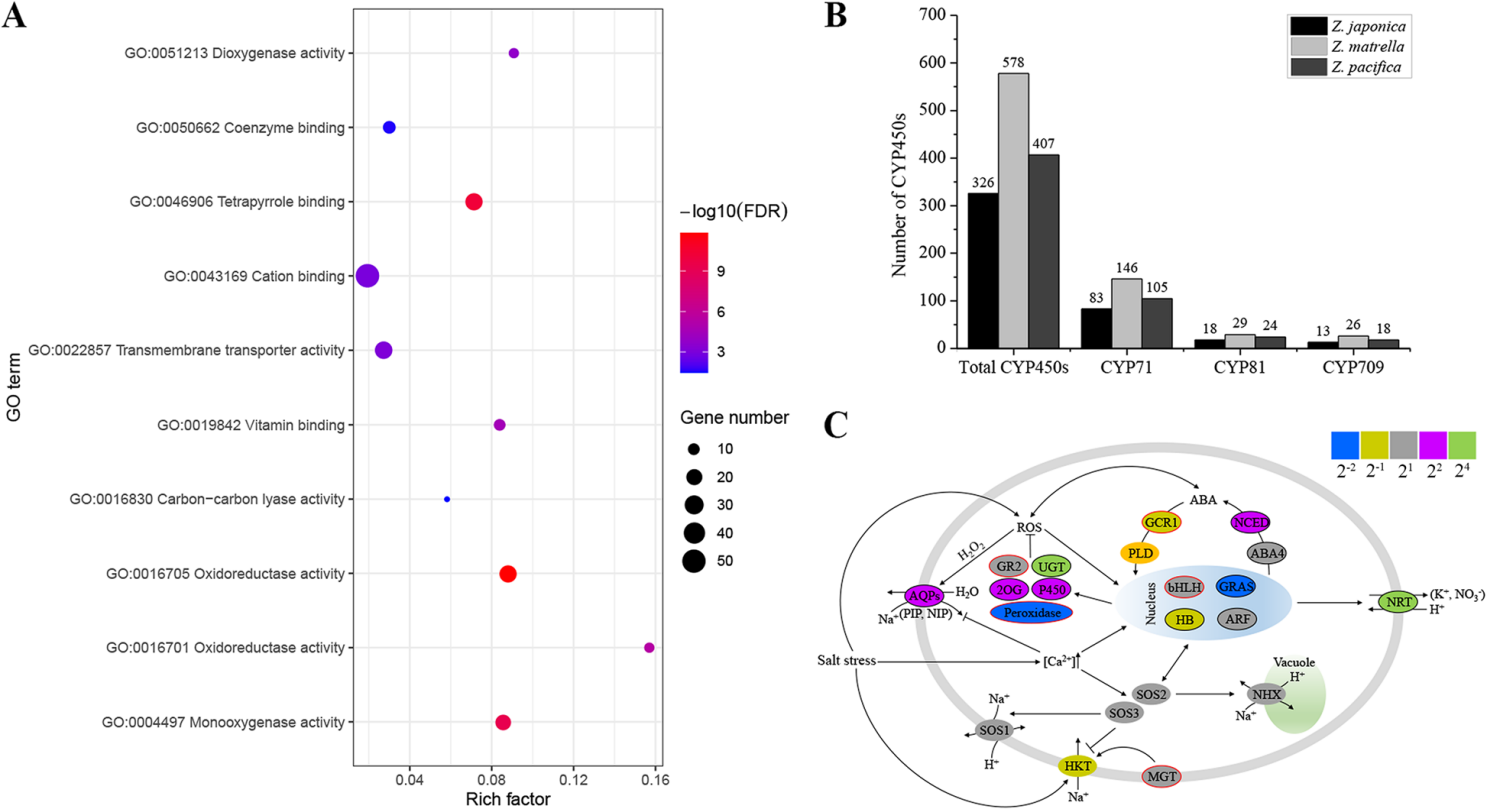
Adaptation of *Z. japonica* to salt stress. (**A**) Enriched GO terms of 68 significantly expanded gene families in the ancestral lineage of three zoysiagrass*.* (**B**) The frequency of CYP450s in three zoysiagrass. (**C**) The positively selected genes (PSGs) and expanded key genes in salt-stress response pathways of *Z. japonica*. Boxes with borders indicate PSGs (red) and expanded (black) *Z. japonica* genes, and the filled colors correspond to their degree of regulation in (FPKM_treatment_/FPKM_control_) at 24 h in root in response to salt stress

To further investigate the evolution of gene families, we identified expanded and contracted gene families. A total of 68 and 117 significantly expanded and contracted gene families in the ancestral lineage for *Zoysia* were identified compared with the other seven plant genomes. The genes from the expanded gene families were mainly enriched in oxidoreductase activity (GO:0,016,705), transmembrane transporter activity (GO:0,022,857) and cation binding (GO:0,043,169) (Fig. 3A and Additional file 7: Table S5), while the 117 contracted gene families were significantly overrepresented in protein phosphorylation (GO:0,006,468) and recognition of pollen (GO:0,048,544) (Additional file 8: Table S6). In addition, the enriched term “oxidoreductase activity” (GO:0,016,705) contains many cytochrome P450 genes, which encode proteins participating in multiple metabolic pathways and playing important roles in multiple processes, particularly roles in stress responses. To assess the function of these genes, we used the seed file of the P450 domain (PF00067) as a query to search the zoysiagrass protein sequences using the cutoff E-value set as 1e-05, and 326, 578 and 407 cytochrome P450 genes were identified in the *Z. japonica*, *Z. matrella* and *Z. pacifica* genomes, respectively (Fig. 3B and Additional file 9: Table S7). Among the 326 cytochrome P450 genes in *Z. japonica*, we found that many probably had functions for salt tolerance. For example, previous study found that *CYP71D8L* enhances tolerance to drought and salt stress by affecting gibberellin (GA) and cytokinin (CK) homeostasis in rice [19]. Among these expanded gene families, there were 83, 146 and 105 *CYP71* genes in *Z. japonica*, *Z. matrella* and *Z. pacifica*, respectively (Fig. 3B). In wheat, constitutively expressing *CYP81D5* enhances salinity tolerance both at the seeding and reproductive stages by accelerating ROS scavenging [20], and there are 18, 29 and 24 *CYP81* genes among the expanded gene families in *Z. japonica*, *Z. matrella* and *Z. pacifica*, respectively (Fig. 3B). Expression of wild-type *CYP709B3* can rescue the salt-sensitive phenotype through an unknown pathway independent of the well-characterized regulator in *A. thaliana* [21], and there are 13, 26 and 18 members of CPY709 genes among the expanded gene families in *Z. japonica*, *Z. matrella* and *Z. pacifica*, respectively (Fig. 3B). Thus, it is likely that some cytochrome P450 genes are responsible for salt tolerance in zoysiagrass. Furthermore, we found that some ABA-related genes involved in ABA biosynthesis were expanded in the *Z. japonica*, *Z. matrella* and *Z. pacifica* genomes (Fig. 3C and Additional file 10: Fig. S3). The NCEDs (9-cis-epoxycarotenoid dioxygenases), which cleave neoxanthin and produce the C15 intermediate xanthoxin, are generally considered to be the first specific and rate-limiting step for ABA biosynthesis (Additional file 10: Fig. S3A). The *Z. japonica*, *Z. matrella* and *Z. pacifica* genomes contain 8, 12 and 8 NCEDs, respectively, while other Poaceae plant species contain 2–7 (Additional file 10: Fig. S3B and C). ABA4 (abscisic acid-deficient 4) and NXD1 (neoxanthin-deficient 1), which are involved in converting violaxanthin into neoxanthin in the ABA biosynthetic pathway, were also expanded in the three zoysiagrasss (Additional file 10: Fig. S3B). These results suggest that the enhanced regulation of ABA biosynthesis may contribute to abiotic stress tolerance in zoysiagrass.

### Positive selection on single-copy genes

Adaptive divergence at the molecular level may also be reflected by an increased rate of nonsynonymous changes within genes involved in adaptation [22, 23]. In collinear regions, we identified 12,642 high-confidence 1:1:1 orthologous genes in the *Z. japonica*, *Z. matrella* and *Z. pacifica* genomes (Additional file 11: Fig. S4). Overall, the *Z. matrella* and *Z. pacifica* genes were very similar, with a mean protein similarity close to 93.11% (Additional file 11: Fig. S4A), while the protein similarity of *Z. japonica* and *Z. pacifica* was approximately 90.83% (Additional file 11: Fig. S4B). The Ka/Ks ratios of nonsynonymous-to-synonymous substitutions for different GO categories of these high-confidence orthologous genes revealed that those genes with elevated pairwise genetic differentiation were primarily enriched in ‘auxin-activated signaling pathway’, ‘response to salt stress’, ‘heat shock protein binding’, and ‘DNA binding transcription factor activity’ (Additional file 12: Table S8), indicating rapid evolution and adaptive divergence in these enriched functions between different zoysiagrass.

To investigate the potential genomic adaptation, we used the branch-site likelihood ratio test to identify positively selected genes (PSGs) in the ancestral lineage of the three zoysiagrasss. Among the 7,495 orthogroups shared by ten species, 963 contained single-copy orthologous genes. As a result, 95 positively selected genes (PSGs) were identified in the zoysiagrass ancestral branch compared to those of other species (Additional file 13: Table S9). A GO functional classification of these PSGs was performed, and some biological processes that were significantly enriched were identified, which were related to membrane lipid metabolic process and glycolipid metabolic process (Additional file 14: Table S10). They included MGT (magnesium transporter), which encodes a plasma membrane-localized transporter protein and has an important role in conferring salt tolerance by enhancing the transport activity of OsHKT1 [24], GCR1 (G-protein-coupled receptor 1), a G-protein signaling component mediating the plant's response to multiple abiotic stresses and interacting with the G protein α subunit GPA1 to regulate abscisic acid signaling [25, 26], and XTH (xyloglucan endotransglycosylase/hydrolase), which is involved in elevating abiotic stress tolerance by maintaining the structural integrity of the cell wall [27]. In addition, oxidoreductases such as peroxidase, 2-oxoglutarate and Fe(II)-dependent oxygenase and glyoxylate reductase (GR2) also showed signs of positive selection.

### Expanded genes and WGD contributed to salt adaptation

**Fig. 4:**
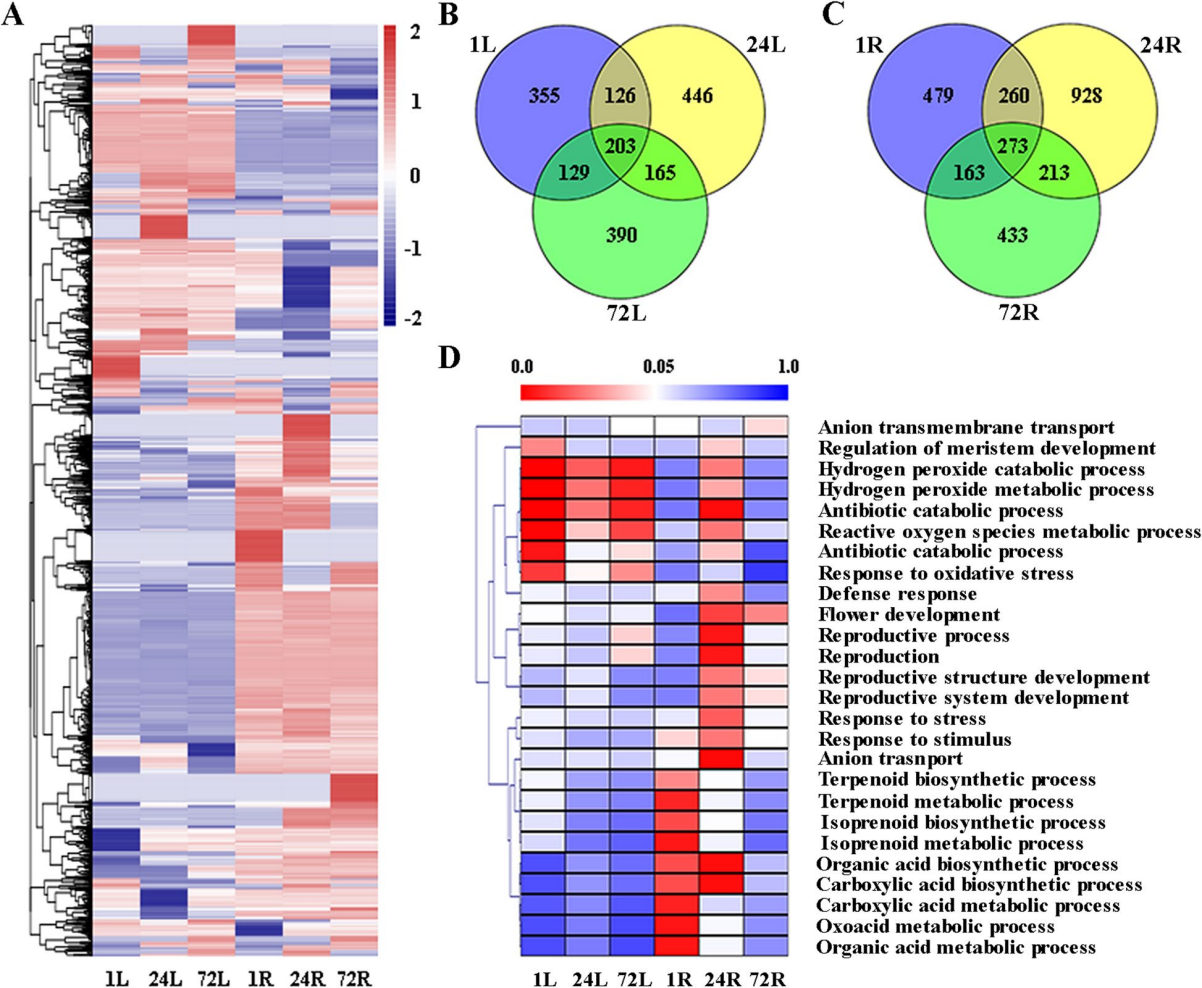
Transcriptomics of *Z. japonica* under salt stress. (**A**) Expression of DEGs identified in leaf and root at each time point. The heatmap was generated from hierarchical cluster analysis of genes. (**B-C**) Venn diagram of the number of DEGs in leaf and root at each time point, respectively. (**D**) Heat maps of significantly enriched pathways in leaf and root during salt stress. The blue and red colours indicate the *P*‐values for significantly enriched pathways for each sample

To further investigate the genetic mechanisms underlying salt tolerance, we performed transcriptomic analysis under salt stress conditions in *Z. japonica* using publicly available data [8]. Differentially expressed genes (DEGs) were identified under salt stress by comparing each time point (1, 24 and 72 h) with 0 h. A total of 813, 940, 887, 1175, 1674 and 1082 DEGs were identified in the 1 h leaf, 24 h leaf, 72 h leaf, 1 h root, 24 h root and 72 h root, respectively (Fig. 4A). Of the 3991 DEGs, the majority were specific to each of the six samples, and only 10 DEGs were shared by all samples (Additional file 15: Table S11). A total of 203 and 273 genes were differentially expressed in salt-stressed leaves and roots, respectively (Fig. 4B and C), and several genes encoding transcription factors, such as AP2/ERF, bZIP, MYB, MADS18 and WRKY, and other genes, such as CYP450, PP2C, CML31 and MEE18, were involved in the response to salt stress (Additional file 16: Table S12). Gene enrichment analysis suggested that many of the DEGs exhibited different regulatory patterns in response to salinity between the two tissues and were mainly enriched in response to oxidative stress, hydrogen peroxide metabolic process and reactive oxygen species metabolic process in salt-stressed leaves, while they were involved in anion transport, organic acid biosynthetic process and reproductive process in salt-stressed roots (Fig. 4D).

**Fig. 5:**
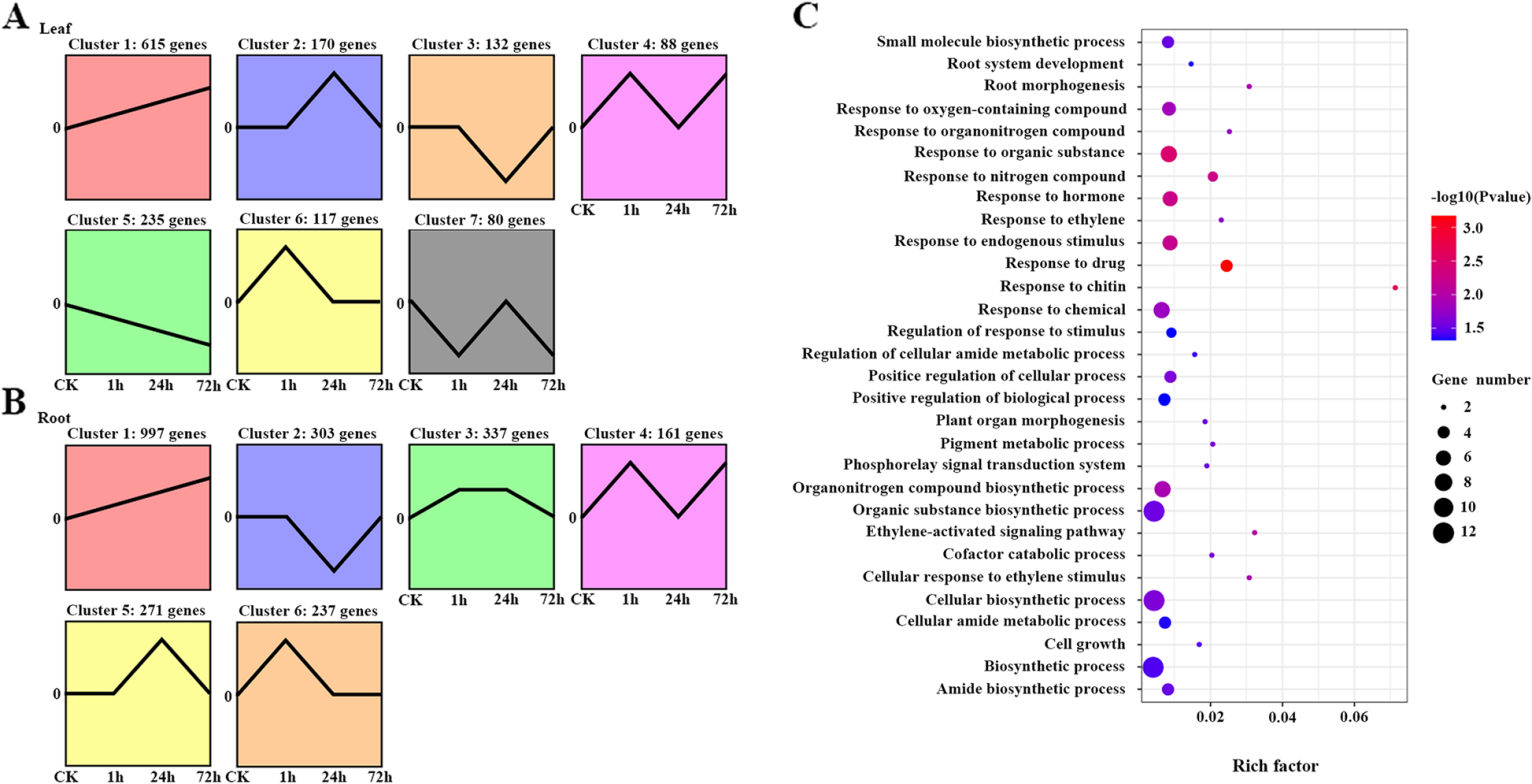
Expression patterns of DEGs and enrichment analysis of the 104 pairs of paralogous genes in *Z. japonica* under salt stress. (**A–B**) Cluster analysis of DEGs displaying a log_2_ fold change of genes during salt stress at 1, 24 and 72 h in leaf and root respectively. The comparisons include control versus control (CK), 1 h versus control (1 h), 24 h versus control (24 h) and 72 h versus control (72 h). (**C**) Enriched GO terms of 104 pairs of paralogous genes in *Z. japonica* under salt stress

Coexpression analysis showed that the 1814 and 2749 differentially expressed genes (DEGs) formed seven and six major clusters in leaves and roots, respectively (Fig. 5A and B). In general, Cluster 1 (615 DEGs) and Cluster 5 (235 DEGs) were the two largest modules containing upregulated and downregulated genes in leaves respectively (Fig. 5A). DEGs of both clusters were induced at all times after salt treatment and were mainly enriched in plant-type secondary cell wall biogenesis, amino acid transport and cofactor catabolic processes in Cluster 1, while they were mainly enriched in response to UV-B, phototransduction and transmembrane transport in Cluster 5 (Additional file 17: Table S13). In root, Cluster 1 (997 DEGs) and Cluster 2 (303 DEGs) were the two largest modules containing upregulated and downregulated genes, respectively, and contained genes for ion and abscisic acid transport, response to stress and abiotic stimulus and response to gibberellin in Cluster 1, while they comprised genes enriched in cell redox homeostasis, regulation of reproductive process and posttranslational protein modification in Cluster 2 (Additional file 18: Table S14). Therefore, the major clusters and DEGs revealed in leaves and roots were different, probably in relation to diverse adaptive mechanisms in different tissues. In addition, the DEGs from Cluster 1 in the roots contained more DEGs derived from the B subgenome (299 vs. 365, χ^2^ test, *P* < 0.05) (Additional file 19: Fig. S5), and DEGs from the A subgenome were enriched in cellular detoxification and secondary metabolic processes, while DEGs from the B subgenome were enriched in ion transport, response to stress and abiotic stimulus, suggesting that the B subgenome of *Z. japonica* contributes to salt tolerance (Additional file 20: Table S15).

We then investigated the expression patterns of expanded and positively selected genes (Additional file 21: Fig. S6). The results showed that some of the expanded genes were induced under salt treatment. Four NRT (nitrate transporter) genes in roots were rapidly upregulated at 1 h and showed a significantly higher relative expression at 24 h, and three GH3 (auxin-responsive GH3 family protein) and five PHT (phosphate transporter) genes were significantly altered by salt stress for at least one time point in the root. *CYP71B23* was significantly upregulated, and *CYP716A1* was downregulated in leaves. *UGT71B6*, 2-oxoglutarate and Fe(II)-dependent oxygenase and MATE efflux family proteins were also induced under salt treatment in leaves or roots (Additional file 21: Fig. S6A). In addition, the expression levels of some positively selected genes, including *MGT10* (magnesium transporter 10), *BRIZ2* (zinc finger family protein) and *MMD1* (PHD-finger domain containing protein), were also increased by salt stress for at least one time point in roots (Additional file 21: Fig. S6B-D). These results provide evidence that expanded and positively selected genes may confer important functions in salt tolerance in *Z. japonica*. Finally, we also examined the expression patterns of paralogous genes in *Z. japonica* under salt stress. We focused on the paralogous genes that have two copies in *Z. japonica* with one synteny copy in *O. thomaeum*, and a total of 1669 paralogous gene pairs were identified. Among them, 1565 pairs of genes showed no DEG, 100 pairs contained one DEG, and 4 pairs had two DEGs in each paralogous gene pair (Additional file 22: Table S16). These 104 pairs of genes with differential expression were enriched in many functional categories related to salt stress, such as response to hormone, response to endogenous stimulus and ethylene-activated signaling pathway (Fig. 5C and Additional file 23: Table S17). These results suggested that many duplicated paralogous genes from the extra WGD event in *Z. japonica* had evolved new functions during its salt adaptation.

## Discussion

Soil salinity severely limits plant distribution and biomass productivity worldwide, and this is becoming an enormous threat because of increasing climatic changes and human activities [1, 2]. Zoysiagrass can resist injury under 1.0% salt solution and is considered to be the most salt-tolerant of the C4 grass species in the family *Poaceae* [7, 11]. In addition, *Z. japonica* and *Z. matrella* are defined as halophytes, suggesting that these species are most likely to have developed more adaptive features to salt stress than other glycophytes. Although some studies have been conducted on the genetic and molecular mechanisms underlying salt tolerance in zoysiagrass [16, 28–31], our current understanding of these aspects of salt tolerance remains limited, especially lacking evidence at the genomic level. In this study, we integrated genomic and transcriptomic analyses to clarify the genomic basis of salt tolerance in zoysiagrass.

Genetic variation and population structure analysis among 248 Zoysia accessions suggested that *Z. matrella* may represent an interspecific hybrid between *Z. japonica* and *Z. pacifica* [15]. However, the genomic evidence for evolutionary relationships among these three zoysiagrasses is still unknown. Phylogenetic analysis revealed that *Z. matrella* had a close relationship with *Z. pacifica* and diverged from each other after the divergence of *Z. japonica* at 3.92 Mya (Fig. 1C). These results provide additional evolutionary evidence for the hypothesis that *Z. matrella* may represent an interspecific hybrid between *Z. japonica* and *Z. pacifica*. The whole-genome duplication or polyploidization events played a important role in genome expansion, evolution and diversification [32–34]. And research has revealed that all the monocots experienced a common WGD event and that Gramineae species shared another common WGD event [35]. In present study, the genome analysis suggested that a shared whole-genome duplication (WGD) event occurred approximately 20.8 Mya for the ancestor lineage of these three zoysiagrasses (Fig. 1D), suggesting that zoysiagrass had an another recent WGD event. In additon, following an allopolypioidy event, a dominant subgenome often emerges with significantly more retained gene and higher homoeolog expression, and the patterns of biased fractionation have been observed in bread wheat [36], *Brassica rapa* [37] and cotton [38]. Besides, some allopolyploids including *Eragrostis tef* [39] and Cucurbita species [40] display no subgenome dominance, suggesting that biased homoeolog fractiontion is not universal. Our results revealtd that there was no significant biased fractionation between the *Z. japonica* subgenome in different tissues and salt treatments (Fig. 2B), it is possible that the allotetraploid formed through a recent hybridization event typically exhibits gene retention with little genome reduction [41, 42] or the genes from the dominant sbugenome replaced their homoeologs from the recessive subgenome, which weakened the patterns of subgenome dominance. Interestingly, although no homologous gene expression bias was detected between the A and B subgenomes, more pathways were involved in the response to abiotic stress, hormones and other stress-response pathways for homologous expression bias from the B subgenome (Fig. 2C and Additional file 4: Table S2), which may reflect the adaptation to adverse environments. The different subgenomes contributing to different characteristics were also founded in allotetraploid cotton, which is A subgenome contribute to fiber improvement, while D subgenomes contribute to wider adaptation [43].

The expansion and contraction of gene families are considered to play important roles in adaptive phenotypic diversification [44]. Examining the expansion or contraction of gene families in depth has been instrumental in understanding functional trait evolution and has been performed in many plant species to illustrate the molecular mechanism of phenotypic adaptation [23, 45, 46]. For example, comparative genomic analysis suggested that the expanded gene families involved in ion and nutrient transport and ABA homeostasis and signaling may contribute to abiotic stress tolerance in quinoa [46]. The expanded cytochrome P450 and chitinase gene families and the jasmonic acid (JA) biosynthetic pathway play an important role in salt tolerance in pistachios [45]. In our study, some stress-related gene families were conserved and expanded in the ancestral lineage of three zoysiagrass species (Fig. 3 and Additional file 10: Fig. S3). Abscisic acid (ABA), which is the central regulator of abiotic stress resistance, allows plants to cope with different stresses [47]. The NCEDs that cleave neoxanthin and produce the C15 intermediate xanthoxin are generally considered to be the first specific and rate-limiting step for ABA biosynthesis. Compared with other species, the NCED gene family was significantly expanded in *Z. japonica*, *Z. matrella* and *Z. pacifica* (Additional file 10: Fig. S3). Cytochrome P450s (CYPs), as the largest enzyme family of plant metabolism, participate in multiple physiological processes and play important roles in various processes, particularly stress responses [45]. Several genomic studies have also reported the expansion features of cytochromes P450, which are considered to contribute to the differences in environmental adaptation [45, 46, 48]. The conserved and expanded cytochromes P450 was also examined in three zoysiagrass species relative to other species (Fig. 3B). In addition to NCED and cytochromes P450, several families of genes involved in ion transport processes were also expanded, including the NRT and PHT families (Fig. 3C). Toxic Cl^−^ ions are expelled from the leaf mesophyll via *SLAH*, pass the stack cell and are ultimately loaded into epidermal bladder cells via NRT transporters, which is an important mechanism for quinoa adaptation to saline conditions [46]. The *PHT1;9* of salt cress participates in root-to-shoot translocation of Pi, and the increased shoot P in transgenic plants promotes K^+^ uptake and translocation to the shoot, which helps to maintain K^+^/Na^+^ homeostasis in shoots under salinity [49]. The expanded NRT and PHT families may contribute to maintaining ion homeostasis in zoysiagrass under various stress environments. Moreover, the transcriptome data showed that some expanded families, including cytochromes P450, NRT and PHT, were significantly induced by salt stress, which further suggested their important roles in the response to salt stress.

Differential expression analysis revealed that the majority of the DEGs were specific to each of the six samples (Fig. 4), suggesting that they exhibited different regulatory patterns in response to salinity between the two tissues of *Z. japonica* under salt treatment. Only 10 DEGs were shared by all six samples, such as transcription factor NAC17 and receptor-like protein kinase 2 (Additional file 15: Table S11). Transcription factors are involved in various biological processes and play crucial roles in the response to biotic and abiotic stresses [50–52]. For example, *NAC3* was induced by drought, high temperature, salinity stress and abscisic acid treatment in rice, and overexpression of *NAC3* resulted in enhanced tolerance to heat and drought stress through modulation of reactive oxygen species [51]. In *A. thaliana*, the R2R3-MYB transcription factor AtMYB49 contributes to salt tolerance by modulating cuticle formation and antioxidant defense [52]. A number of transcription factors, including AP2/ERF, bZIP, MYB, MADS18 and WRKY, were induced in salt-stressed leaves or roots, which suggested that these transcription factors might be involved in the response to salt stress in *Z. japonica* (Additional file 16: Table S12). Whole-genome duplications or polyploidizations provide functional innovations through different mechanisms and play critical roles in plant adaptations to stressful habitats [32–34]. In this study, a total of 104 pairs of duplicate genes that had at least one DEG in each pair were identified and enriched in response to hormone and ethylene-activated signaling pathways, suggesting that these duplicate genes have evolved new functions and played an important role in response to salt stress (Fig. 5C and Additional file 23: Table S17).

## Conclusions

In this study, we examined the genomic signatures of adaptive evolution for zoysiagrasses, including *Zoysia* underwent a genus-specific whole-genome duplication (WGD) event after divergence from the *O. thomaeum* lineage, and stress adaptation of zoysiagrasses is likely attributable to the expanded cytochrome P450 and the abscisic acid (ABA) biosynthesis-related gene families. By transcriptomic analysis, we further found that many duplicated genes from the extra WGD event exhibited distinct functional divergence in response to salt stress, suggesting that this WGD event contributed to strong resistance to salt stress. Our results provide an important and valuable basis for understanding zoysiagrass adaptation to salt stress and facilitate the genetic improvement for molecular breeding in zoysiagrass.

## Methods

### Genomic data

A total of ten sequenced plant genomes, including nine monocots (*Brachypodium distachyon, O. sativa, S. bicolor, S. italica, Zea mays, O. thomaeum, Z. japonica, Z. matrella, Z. pacifica*) and one dicot model *A. thaliana*, were subjected to comparative genomic analysis. This includes three zoysiagrasses which can resist injury under 1.0% salt solution and are considered to be the most salt tolerant of the C4 grass species in the family *Poaceae* [7, 11]. In addition, *Z. japonica* and *Z. matrella* are defined as halophytes while *O. sativa, S. bicolor, S. italica, Zea mays* and *A. thaliana* are defined as glycophytes [11, 12]. To investigate the structure of the subgenome and the patterns of subgenome differentiation and dominance, *O. thomaeum* which has the closest phylogenetic relationship with zoysiagrass was used in this study. Genome data of *Z. japonica, Z. matrella* and *Z. pacifica* were downloaded from the *Zoysia* Genome Database (version 1.0, http://zoysia.kazusa.or.jp/) [9], and the other genome data were mainly downloaded from Phytozome (version 13) [53].

### Gene family clusters

The longest translation form of the protein-coding genes from ten plant species was selected to represent each gene, and stretches of genes coding shorter than 50 aa or containing stop codons more than 20% were filtered out. All filtered protein sequences of these species were compared with each other using BLASTP (version 2.5.0) with an E-value < 1e-5 [54], and sequence pairs with a percentage identity of at least 30% and query coverage of at least 30% were clustered into orthologous groups using OrthoMCL software (version 2.0.9) with an inflation parameter set as 1.5 [18]. Species-specific and *Zoysia* genus-specific genes were identified, and orthogroups with single-copy genes shared by all ten genomes were retained for further analyses.

### Phylogenetic tree construction and subgenome identification

Protein sequences from 963 single-copy gene families across all ten species retrieved from the OrthoMCL (version 2.0.9) [18] results were used for phylogenetic tree construction. MAFFT (version 7.407) [55] was used to generate multiple sequence alignment for protein sequences of each single-copy family with default parameters, and then, alignments were trimmed using Gblocks (version 0.91b) [56]. The alignments of each family were concatenated into an alignment supermatrix to construct a species tree using RaxML under the best protein substitution model ‘PROTGAMMALGX’ with 1000 bootstraps [57], with *A. thaliana* as an outgroup according to the selection results of outgroup in *Achnatherum splendens* [58], *Miscanthus lutarioriparius*[59], *Echinochloa crus-galli* [60] and *Cenchrus purpureus* [61]. In addition, Bayesian Evolutionary Analysis Sampling Trees (BEAST) (version 2.5.0) [62] was used to estimate species divergence times using an uncorrelated relaxed clock with the Blosum62 substitution model and Yule speciation process and based on a split between *O. sativa* and *B. distachyon* (mean: 46.0 MYA. Std dev: 1.0 MYA) [63] as the calibration times. The Markov chain Monte Carlo was run for 100,000,000 generations with sampling every 1000 generations. The phylogenetic tree was visualized using FigTree (version 1.4.3) (http://tree.bio.ed.ac.uk/software/figtree/).

*Z. japonica* genes were aligned to each other to identify paralogs within the genome, and MCScanX was used to construct genomic synteny blocks between *Z. japonica*-*O. thomaeum* and *Z. japonica*-*Z. japonica* using the default parameters [64]. Then, the subgenome of *Z. japonica* was identified based on the synteny relationships between *Z. japonica* pseudomolecules and *O. thomaeum* chromosomes.

### Gene family expansion and contraction

Gene family expansion and contraction analyses for each species were performed using CAFÉ (version 4.2) [65]. Only gene families containing at least one gene in no less than two species and fewer than 100 genes for one or more species were retained for the analysis. An “expanded and contracted gene family” with both an overall *P* value (family-wide P value) and an exact *P* value (Viterbi method) ≤ 0.01 was defined as a “significantly expanded and contracted gene family”. Gene Ontology (GO) enrichment analyses for these expansion and contraction gene families were performed using Bioconductor software package topGO in R programming language (version 4.0.3) [66], and the scripts were listed in supplementary data (Additional file 24).

### Whole-genome duplication and evolution analyses

The protein sequences from *Z. japonica, Z. matrella* and *Z. pacifica* were subjected to an all-against-all BLASTP (version 2.5.0) search with an *E* value cutoff of 1e-10. Then, syntenic blocks within each species were detected using MCScanX with default parameters, and at least five genes were required to define synteny blocks [64]. To estimate whole-genome duplication (WGD) events, the third codon transversions within these fourfold degenerate sites (4DTv) of paralogous genes within *Z. japonica, Z. matrella* and *Z. pacifica* were calculated using in-house Perl scripts, while the synonymous substitutions per synonymous site (*K*s) were calculated by PAML (version 4.9 h) using the YN00 YN model [67]. The distribution of the 4DTv distance and all Ks values were plotted via R software (version 4.0.3) using the ggplot2 package. The peak Ks values were converted to the divergence following the formula *T* = Ks/2λ by using an average substitution rate of 6.5e-9 for grasses to infer speciation of WGD events that occurred during the evolutionary history [68].

### Identification of positively selected gene families

To identify potential positively selected genes (PSGs), gene families of ten plant species (*B. distachyon*, *O. sativa*, *S. bicolor*, *S. italica*, *Z. mays*, *O. thomaeum*, *Z. japonica*, *Z. matrella*, *Z. pacifica*) were retrieved from the OrthoMCL (version 2.0.9) [18] results, and a total of 963 single-copy gene families which kept 1:1:1:1:1:1:1:1:1:1 across all used species were used for further identification of PSGs. Based on the MAFFT (version 7.407) alignment sequences, the reverse-translated amino acid alignments to the corresponding codon-based nucleotide alignments were performed using PAL2NAL (version 14.0) [69], and then, the conserved alignments of each single-copy gene family were extracted by Gblocks (version 0.91b) [56] and used for further identification of PSGs. The position selections in *Z. japonica, Z. matrella* and *Z. pacifica* were tested under the reconstructed phylogenomic tree by PAML (version 4.9 h) [70]. For example, likelihood ratio tests (LTRs) based on the branch-site model of Codeml were conducted to detect potential PSGs in *Z. japoncia*, with *Z. japoncia* set as the foreground branch and the other nine plant species as background branches according to the previously described method [71]. The PSGs in *Z. matrella* and *Z. pacifica* were also identified by the same method. In addition, to investigate the potential genomic adaptations in the ancestral lineage of *Zoysia*, the PSGs in the ancestral lineage of *Zoysia* were also identified using three zoysiagrass (*Z. japonica, Z. matrella* and *Z. pacifica*) set as the foreground branch and the other plant species as background branches. Finally, the functional enrichment of these PSGs in GO terms was estimated using topGO [66], with the 963 single-copy genes as the background gene set. The scripts were listed in supplementary data (Additional file 24).

### Differential expression analysis and homologous expression bias

To examine genome-wide responses to salt stress, RNA-Seq clean reads (three biological replicates) from a salt-tolerant *Z. japonica* Steud. Z011, which was subjected to salt treatment and sampled at 0, 1, 24 and 72 h were downloaded from the SRA database (BioProject accession: PRJNA559944) [8]. TopHat2 software (version 2.1.1) was used to map the transcriptome reads to the reference *Z. japonica* genome, and FPKM values were estimated using Cufflinks software (version 2.2.1) [72]. Finally, the differential expression analysis for the different sample comparisons was performed by Cuffdiff, and those genes whose *P* value < 0.05 and |log2 (FoldChange)|≥ 1 were defined as significantly differentially expressed genes [72]. Cluster analysis of DEGs was performed using the OmicShare tools (http://www.omicshare.com/tools), and genes in each cluster were subjected to GO enrichment analysis.

MCScanx was used to identify the homologous gene pairs between the A and B subgenome with default parameters, and then the syntenic gene pairs were used for homologous expression dominance analysis. Syntenic gene pairs with |A_subgenome/ B_subgenome|≥ 2 or ≤ 0.5 were defined as dominant gene pairs, and those genes with homologous expression bias toward the A or B subgenome were subjected to enrichment analysis by the topGO package [66], and the scripts were listed in supplementary data (Additional file 24).

## Supplementary Information

**Additional file 1.**

**Additional file 2.** 

**Additional file 3.**

**Additional file 4.**

**Additional file 5.**

**Additional file 6.**

**Additional file 7.**

**Additional file 8.**

**Additional file 9.**

**Additional file 10.**

**Additional file 11.**

**Additional file 12.**

**Additional file 13.**

**Additional file 14.**

**Additional file 15.**

**Additional file 16.**

**Additional file 17.**

**Additional file 18.**

**Additional file 19.**

**Additional file 20**.

**Additional file 21.**

**Additional file 22.**

**Additional file 23.**

**Additional file 24.**

## Data Availability

All data supporting the findings of this study are available within the paper and within its supplementary data.
