# Supplementary figures and images for: Comparative genomics reveals the molecular mechanism of salt adaptation for zoysiagrasses
Source: BMC Plant Biol. 2022 Jul 21;22:355. doi: 10.1186/s12870-022-03752-0 (PMC9306052; doi:10.1186/s12870-022-03752-0)

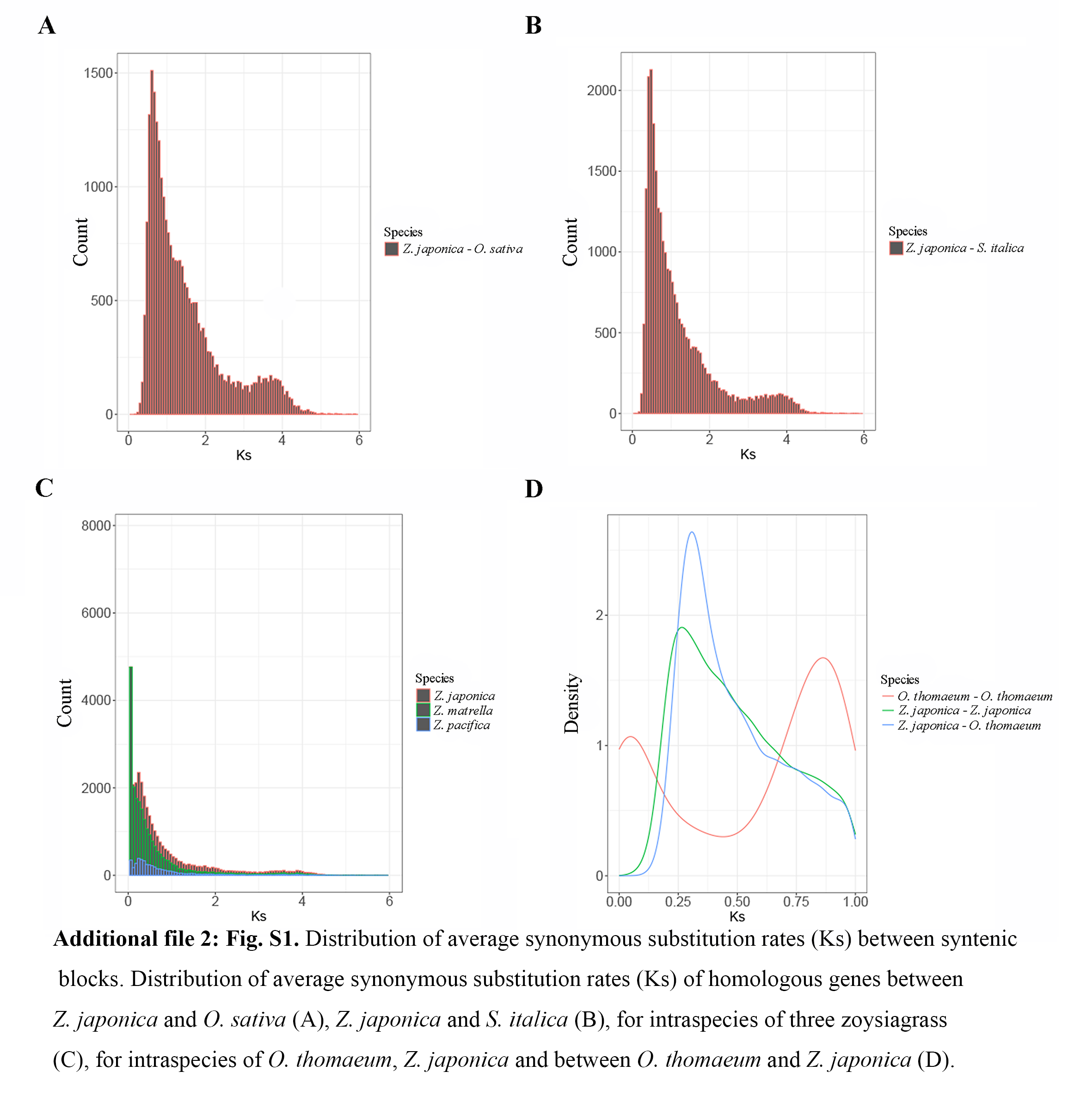

Supplement: Supplementary file 2 — Additional file 2. [file 12870_2022_3752_MOESM2_ESM.tiff]

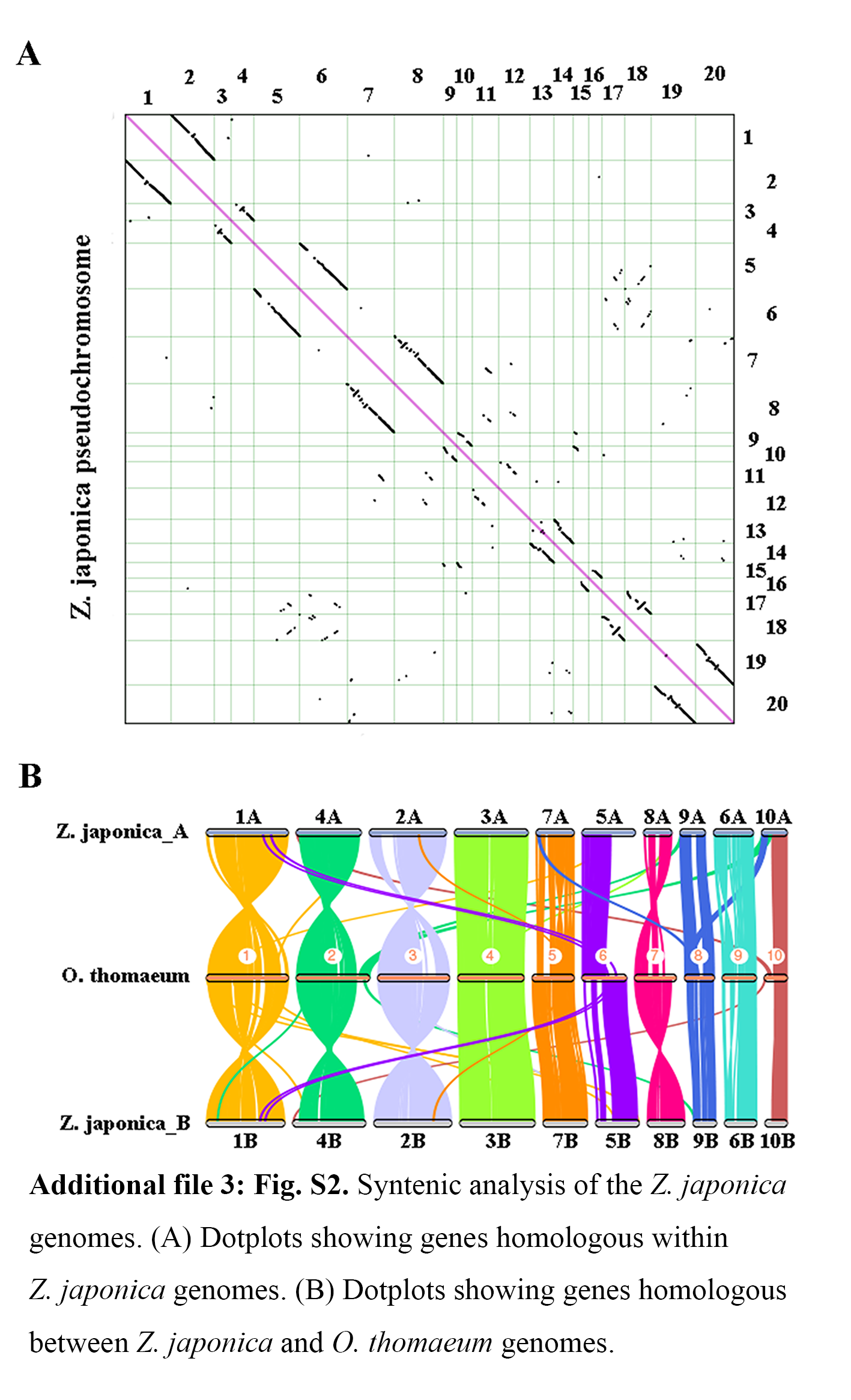

Supplement: Supplementary file 3 — Additional file 3. [file 12870_2022_3752_MOESM3_ESM.tiff]

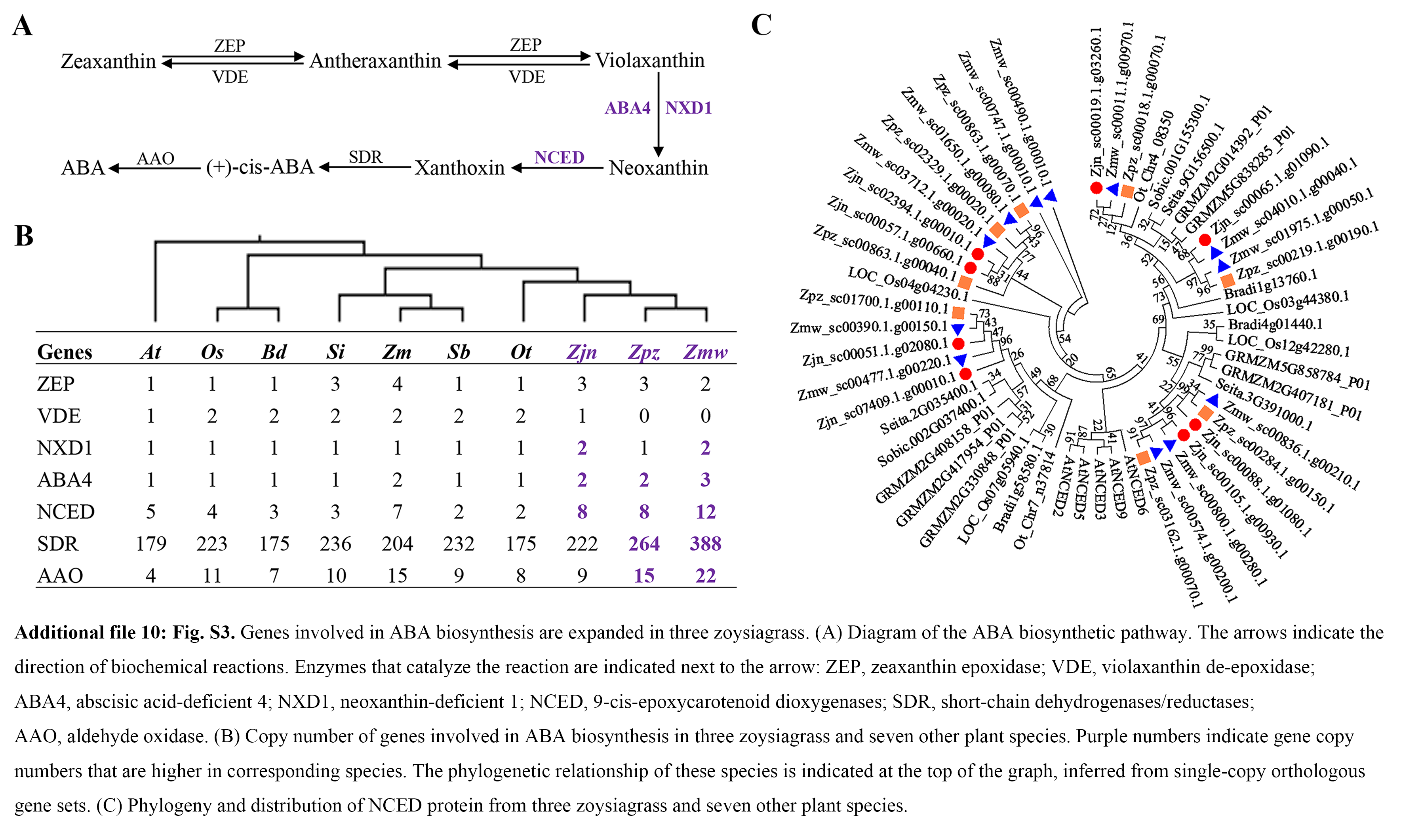

Supplement: Supplementary file 10 — Additional file 10. [file 12870_2022_3752_MOESM10_ESM.tiff]

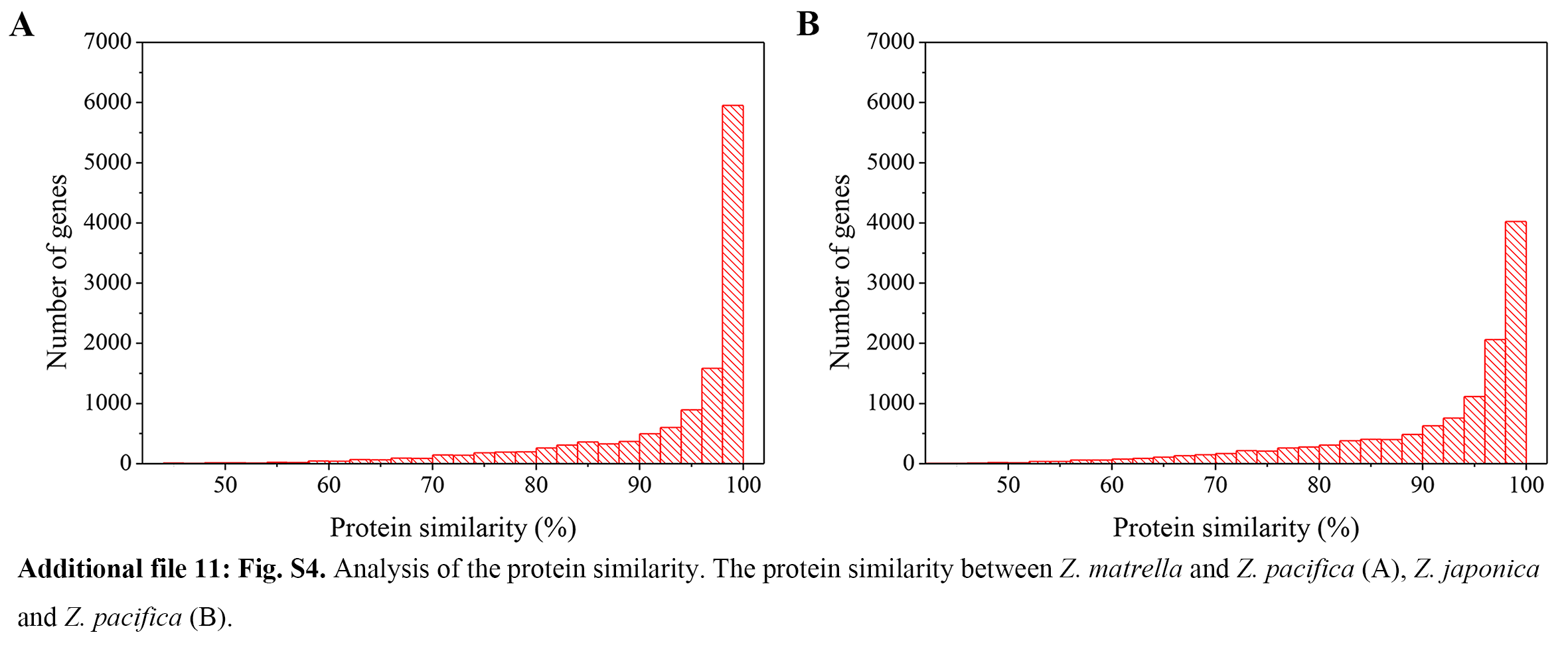

Supplement: Supplementary file 11 — Additional file 11. [file 12870_2022_3752_MOESM11_ESM.tiff]

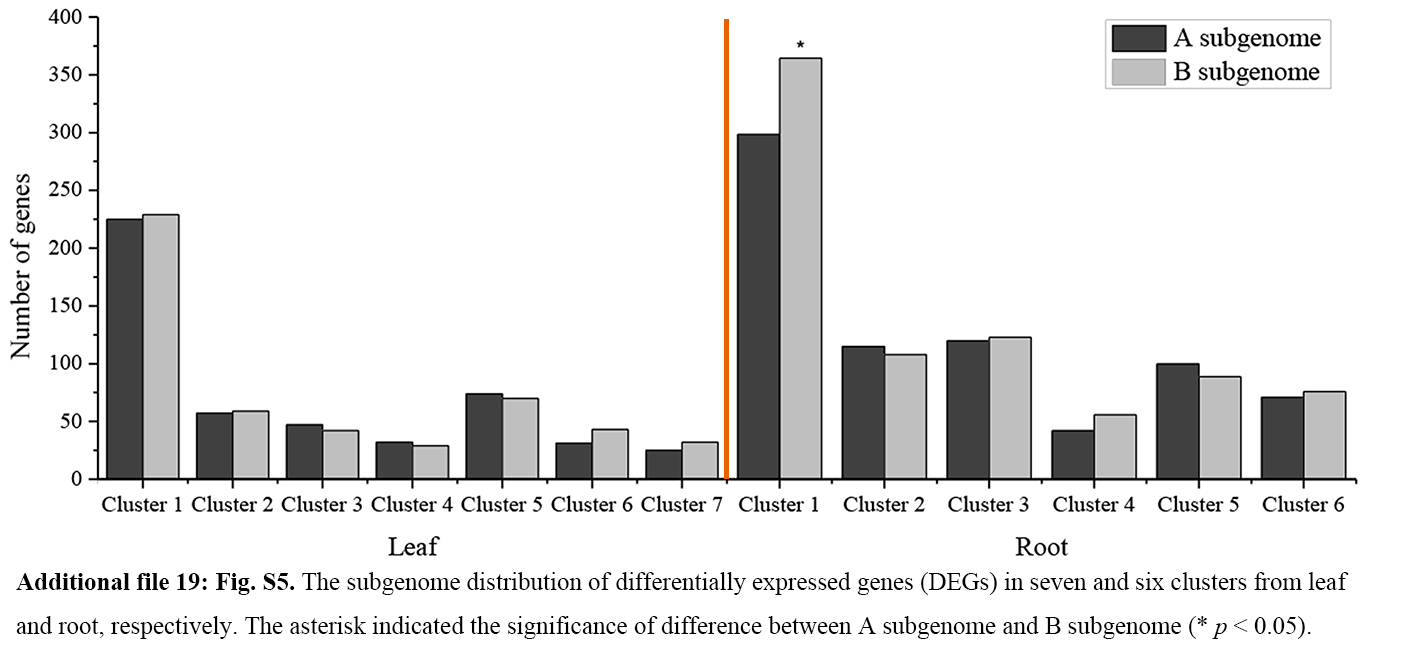

Supplement: Supplementary file 19 — Additional file 19. [file 12870_2022_3752_MOESM19_ESM.tiff]

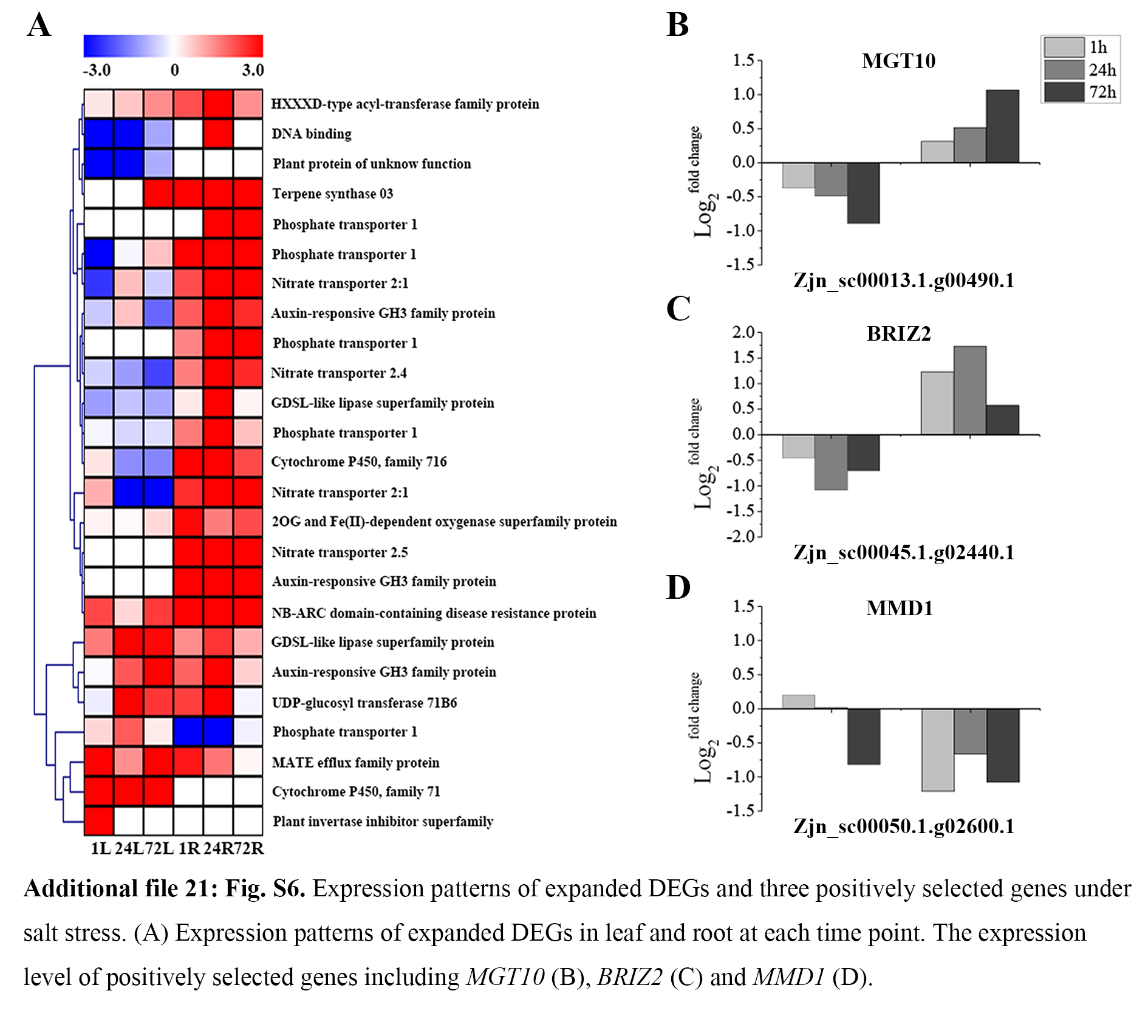

Supplement: Supplementary file 21 — Additional file 21. [file 12870_2022_3752_MOESM21_ESM.tiff]
