# Supplementary material for: Comparative genomics reveals the molecular mechanism of salt adaptation for zoysiagrasses
Source: BMC Plant Biol. 2022 Jul 21;22:355. doi: 10.1186/s12870-022-03752-0 (PMC9306052; doi:10.1186/s12870-022-03752-0)
Supplement: Supplementary file 24 — Additional file 24. [file 12870_2022_3752_MOESM24_ESM.docx]

**Additional file 24:** The R scripts used for plotting or analyzing data.

1. The scripts used for Bioconductor software package topGO in R programming language.

>R

>library("topGO")

>library("Rgraphviz")

>geneid2GO<-readMappings(file="backgroud.txt")

>genenames<-names(geneid2GO)

>gene_data<-read.table("geneList.txt")

>gene_id<- gene_data[,1]

>genelist <- factor(as.integer(genenames %in% gene_id))

>names(genelist) <- genenames

>GOdata <- new("topGOdata", allGenes = genelist, ontology = "BP", annot = annFUN.gene2GO, gene2GO = geneid2GO)

>resultFisher <- runTest(GOdata, algorithm = "classic", statistic = "fisher")

>sig.tab<- GenTable(GOdata,Fis=resultFisher,topNodes=500)

>write.csv(sig.tab,"topGO_BP.csv")

2. The scripts used for plotting by ggplot2 in R programming language.

>R

>library("ggplot2")

>data<-read.table("gene_expression_data.txt", header=T)

>pdf("gene_expression_data.pdf")

>ggplot(data,aes(x=log))+geom_histogram(binswidth=1,color='white',breaks=seq(-10,10,1))+theme_bw()+theme(panel.grid.major=element_line(colour=NA))+theme(panel.grid.minor=element_line(colour=NA))

>dev.off()

3. The scripts used for plotting by pheatmap in R programming language.

>R

>library("pheatmap")

>data=read.table("gene_expression_data.txt",sep="\t", header=T,row.names=1)

>pdf("gene_expression_data.pdf")

>pheatmap(data,show_rownames=F,scale="row",cluster_cols=F,color=colorRampPalette(c("navy","white","firebrick3"))(50),cellwidth=40)

>dev.off()
